# Biomarker robustness reveals the PDGF network as driving disease outcome in ovarian cancer patients in multiple studies

**DOI:** 10.1186/1752-0509-6-3

**Published:** 2012-01-11

**Authors:** Rotem Ben-Hamo, Sol Efroni

**Affiliations:** 1 The Mina and Everard Goodman Faculty of Life Science, Bar Ilan University, Keren Hayesod St., Ramat-Gan, 52900, Israel

## Abstract

**Background:**

Ovarian cancer causes more deaths than any other gynecological cancer. Identifying the molecular mechanisms that drive disease progress in ovarian cancer is a critical step in providing therapeutics, improving diagnostics, and affiliating clinical behavior with disease etiology. Identification of molecular interactions that stratify prognosis is key in facilitating a clinical-molecular perspective.

**Results:**

The Cancer Genome Atlas has recently made available the molecular characteristics of more than 500 patients. We used the TCGA multi-analysis study, and two additional datasets and a set of computational algorithms that we developed. The computational algorithms are based on methods that identify network alterations and quantify network behavior through gene expression.

We identify a network biomarker that significantly stratifies survival rates in ovarian cancer patients. Interestingly, expression levels of single or sets of genes do not explain the prognostic stratification. The discovered biomarker is composed of the network around the PDGF pathway. The biomarker enables prognosis stratification.

**Conclusion:**

The work presented here demonstrates, through the power of gene-expression networks, the criticality of the PDGF network in driving disease course. In uncovering the specific interactions within the network, that drive the phenotype, we catalyze targeted treatment, facilitate prognosis and offer a novel perspective into hidden disease heterogeneity.

## Background

Cancer is a disease of genomic alterations: changes in DNA sequence, epigenetic aberrations in DNA methylation and genomic variations in copy number together underpin the development and progression of human malignancies [1]. Causing more deaths than any other gynecological cancer, epithelial ovarian cancer had an estimated 21,550 new cases and 14,600 deaths in the United States in 2009 [2]. Ovarian cancer strikes silently, revealing no obvious symptoms until late in its course, leading to late stage diagnosis [3]. The best therapy for ovarian cancer remains undetermined. Patients with well-differentiated tumor stages IA, IB show good prognosis and surgery is sufficient, but for patients with more advanced stages, optimal treatment after surgery has not been completely defined; most patients receiving aggressive therapy display poor prognosis, questioning the real impact of treatments on the biology of the tumor [4]. A better understanding of the biology of advanced ovarian cancer may help improve the treatment for patients with more advanced tumor stages. Identification of cellular factors that drive the prognosis may provide a key to novel treatment. [5]. Systems biology approaches hold the promise of substantially improving the current state-of-the-art in medicine by clarifying distinctions between multiple disease states and enabling the underlying molecular causes of a disease to be identified [6-8].

One of the most comprehensive efforts in molecular characterization of cancer in general and ovarian cancer in particular is The Cancer Genome Atlas (TCGA) [1]. The types of data provided through TCGA, for over 500 patients, are expression abundance through microarrays, DNA methylation and copy number variation data. DNA methylation plays an important role in the development of cancer and other diseases owing to its ability to control and silence gene expression through the interaction of methylcytosine binding proteins with other structural components of chromatin, which makes DNA inaccessible to transcription factors through histone deacetylation and chromatin structure changes [9-11]. Somatic copy number variations are extremely common in cancer. Deletions and amplifications contribute to alteration in the expression of tumor suppressor genes and oncogenes. By studying these changes and their versatility, we can find targets for sophisticated therapeutics approaches [12,13].

In this work, we analyzed methylation, copy number and gene-expression data for 511 ovarian cancer patients from The Cancer Genome Atlas database, and gene-expression data from two additional datasets obtained from the Duke University Medical Center [14,15], to determine molecular concomitants of disease outcome. As a first step, we determined the list of genes whose expression levels stratify patients into groups with distinct prognoses. However, when we verified the molecular behavior of these genes in other, unrelated, datasets, the gene signature obtained was utterly unsuccessful in achieving prognostic stratification. In addition, we performed gene set signature analysis in order to find sets of genes whose expression patterns correlated with survival, no overlapped signature was found. We therefore addressed the issue from a different perspective and utilized well-documented connectivity and hierarchy of signaling networks in cells to see if modifications in network behavior could be more closely associated with phenotype than the simple expression of single genes. The results we show here demonstrate that such network modifications indeed stratify patient prognoses according to the molecular characterization of the tumor.

Further, and perhaps most importantly, the specific pathway we highlight as network signature can be carried over to new datasets. That is, the same network behavior associates patients with outcome, regardless of specific batches of experimental procedures. Merging datasets from different studies bridges biases, leads to identification of robust survival factors [16] and eases concerns about the instability of mRNA data [17,18]. Applying tests that predict clinical outcome for patients on the basis of RNA abundance in their tumors is likely to affect patient management increasingly, heralding a new era of personalized medicine [7].

The single gene approach has proven useful in different types of cancer. Established research has shown (e.g.) the connection between MYC and prognosis outcome. High expression levels of MYC correlate closely with poor prognosis in many types of cancers [19-21]. It has been demonstrated that MYC alone can stratify patient groups and it shows a significant p-value in a Kaplan-Meier analysis. Here, however, we found that the single gene approach does not sustain ratification in multiple datasets. In contrast, we demonstrate the ability of a molecular network to serve as a biomarker. By identifying the particular subnetworks that are targeted by genomic aberrations and by demonstrating their phenotypic power through their ability to stratify patient groups, we come closer to identifying a biological process that drives the disease. We emphasize that within the network we identify here; it is not possible to detect single molecules on which phenotypic stratification can be based. Only the combined effect of the relationships among the genes, the measure of their co-dependency through the different pathway metrics we use, drives the phenotypic classification.

## Results and discussion

Kaplan-Meier (KM) survival analysis enables quantifiable metrics to be associated with disease outcome. KM analysis, a well-established method, is often used in clinical and basic research to identify biomarkeres that may improve survival rates. In ovarian cancer datasets, owing to the disease course, other phenotypes (stage, pharmaceutical regiment, environmental parameters, etc.) are usually absent, and disease outcome is often the only strong phenotype available.

The work presented here was performed in three manners: single-gene based, gene-set based and network based.

### Genome wide, single gene based survival analysis

For the single-gene based approach, we retrieved mRNA expression levels for the collection of genes sampled via microarrays used in TCGA (see further details in Methods). We then iterated across the list of 22,777 genes represented on the array.. Each gene was classified using K-means clustering into two groups (K = 2) according to its expression levels. Thus, low expression levels of a specific gene would affiliate patients to one group, while higher expression levels would affiliate them to the other. We thereby generated unique patient groups, per each gene. Next, we used the classification data along with the clinical outcome data to generate 2,2277 KM curves. That is, the gene-expression-based classification into distinct groups was used as the basis for the KM curves and associated p-values. The result of this genome-wide process was a set of p-values, one for each of the genes covered by the microarray (all known genes in the human genome). 1634 genes were found to have significant log-rank p-value in patient stratification. The lists of genes and their corresponding p-values are given in Additional file 1 Table S1.

This procedure was performed first on the TCGA dataset and subsequently on the two additional datasets (Duke set #1: 105 significant genes, and Duke set #2: 249 significant genes). The reason for the repeated procedure was to find a robust set of genes, able to stratify survival in each of the unrelated datasets. 11 genes overlapped between the TCGA dataset and Duke set #1, 16 genes overlapped between the TCGA set and Duke set #2, and only four genes overlapped between Duke #1 and Duke #2 sets.

**Figure 1:**
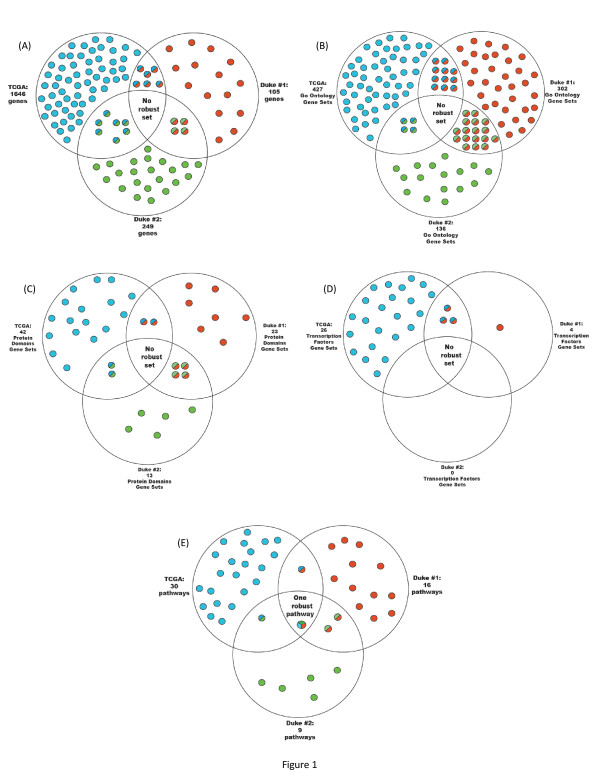
**Venn diagrams demonstrate the overlap/lack of overlap between prognostic biomarkers**. (a) Blue circles stand for the genes identified as significant in stratifying patients into survival groups in TCGA. Red circles are genes identified in Duke set #1 and green circles are genes identified in Duke set #2. The shared colored circles are genes shared between datasets. While some genes are shared between two groups, none is shared among all three datasets. (b-d) Venn diagrams for the gene set signature analysis. In contrast, (e) shows the same analyses performed via pathway metrics. One pathway (PDGF signaling pathway) is shared among the three datasets and demonstrates the robustness of the pathway approach.

Although we could find specific sets of genes with significant p-values in each dataset (see Figure 1a), these gene sets do not overlap across all three datasets. Not even one gene within the gene sets demonstrated robustness across multiple studies.

Gene set enrichment analysis (GSEA) has become a conventional tool for analyzing gene-expression microarray results. It looks at groups of genes and tries to determine whether the members of the group distribute randomly throughout the entire reference list [22]. GSEA is used here to test whether the genes we found in each dataset randomly distribute among all 579 pathways or enrich specific pathways. We used GSEA on the sets of genes we found to stratify prognosis.

Contrary to the gene-set based analysis, in which the analysis focused on finding sets of genes that their combined expression values could stratify the patients into survival groups, here we focused on the entire collection of significant genes in every dataset in order to find enrichment to pathways. This was done in order to reject any biases in the single gene analysis, meaning to verify that the genes found in all three sets in the single gene analysis do not enrich the same pathways.

We found that of the 1,646 genes identified via the TCGA dataset, 51 pathways were enriched and had significant p-values. The 105 genes in the second dataset significantly enriched 24 pathways. Out of the 249 genes in the third dataset, 16 pathways were identified. Again, the intersection of significantly enriched pathways from the three datasets resulted in an empty set. This result strengthens our hypothesis that single-gene-expression levels miss a valuable perspective on the complete process.

### Interactome-Wide, gene set based survival analyses

Discovering biologically meaningful gene patterns is highly important in analyzing genome-wide transcription profiles. In order to identify transcriptional signature that could predict survival rates we used the BRB-Array Tool.

The BRB-Array Tool is an integrated software for the comprehensive analysis of DNA microarray experiments developed at NCI, Biometric Research Branch, Division of Cancer Treatment and Diagnosis [23]. A Gene Set Expression Comparison kit is part of the BRB-Array Tool intended to find meaningful patterns in the data. This analysis enables us to find gene sets of transcription factor (TF) targets, gene sets containing genes whose protein products share the same protein domains, and gene sets with the same GO ontology annotation [24]. Using this analysis, we looked for sets of genes whose expression correlated with patient survival. Goeman's Global Test, which was used here to determine significance, is a score test for the association of the expression profile of a gene set with survival time. Using this test, it can be determined whether the global expression pattern of a group of genes is significantly related to the clinical outcome [25].. This analysis was performed on all three datasets in order to find gene sets that significantly correlated with survival. As in the single gene analysis, we Identified here as well sets of genes that significantly correlated with survival in each dataset, but none of them overlapped between the three datasets. Figure 1b-d demonstrates the results from (b) the Go Ontology, (c) Protein Domains and (d) Transcription Factors gene set analyses.

### Interactome-Wide, pathway based survival analyses

The third approach was to utilized network graph structure. For that, we applied methods for merging expression data with network knowledge [26]. These methods quantify expression behavior in specific sub-networks (i.e. specific pathways or any other defined sub-network) and produce two metrics: network activity and consistency. In brief, a pathway's activity is a measure of how likely the interactions within a pathway are to be active in a specific sample. A pathway consistency is a measure of the compatibility between gene-expression abundance in that sample and molecular description as detailed in the pathway's graph (meaning is the pathway behavior is consistent with the graph structure). Further details are given in the Methods section and in [26].

To apply this network-based methodology, we used The PathOlogist [27] which is an automated Matlab tool that uses gene-expression data(RMA levels) to deduce pathway metrics. Each sample was thus re-represented using its pathway metrics. This representation assigns 579 pathway metric scores (a score for each pathway in the database) to each sample. Interaction and pathway information was obtained from The National Cancer Institute's Pathway Interaction Database (PID) [28]. We then clustered every pathway into two group (according to pathway expression levels) using K-means clustering and iterated across the set of samples,, to assign KM p-values for each of the pathways in order to identify pathways and on the basis of their expression levels we can stratify the patients into two survival groups. This procedure allowed us to rank each pathway, in a similar fashion to the ranking we performed per each gene and for the sets of genes. This entire collection of pathways and genes and their p-values is available in Additional file 1 Table S1 and Additional file 2 Table S2.

We then validated this set of pathways within the two additional data sets used previously [14,15]. Following the same procedure, we found, for every dataset, a set of pathways that stratify prognosis. These multiple computational procedures provide us with three sets of pathways, one for each dataset. Yet the results here were very different from those in the single-gene-based and the gene-set-based approaches. When we intersected the three pathway sets, we found one significant pathway that prevailed across the multiple data sets. Again, the pathway was chosen for its statistical strength in prognosis stratification (survival analyses). Yet no individual gene member by itself showed any statistical power in survival analyses. The combined effect of transcriptional dependence, as expressed by the PDGF signaling pathway, provides this statistical power. The PDGF signaling pathway (Biocarta) showed consistent behavior across all data sets and was the most powerful biomarker in its ability to stratify prognosis very significantly. Figure 1e demonstrates the results.

#### PDGF signaling pathway

**Figure 2:**
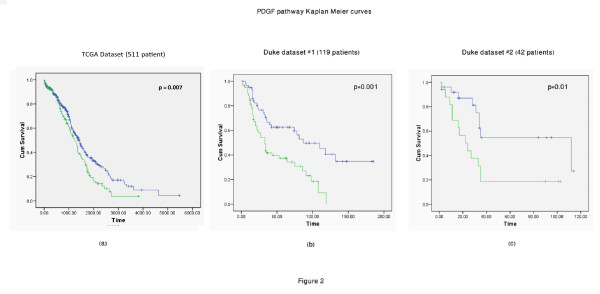
**Kaplan-Meier curves generated according to values of the PDGF pathway**. Panel (a) shows the KM curve generated using the TCGA dataset. Panels (b) and (c) show curves from Duke Dataset #1 and #2 respectively. Across the three panels, Group1 (blue line), which is affiliated with better prognosis, shows lower pathway activity values and Group2 (green line) shows higher pathway activity values. The affiliation of pathway metric levels with prognosis is highly robust in this case, as it shows low p-values and consistent behavior across datasets.

**Figure 3:**
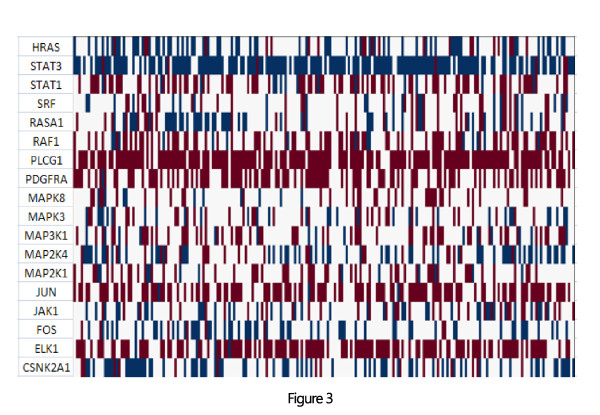
**CNV HeatMap of alteration in gene members of the PDGF signaling pathway across 511 patients from the TCGA database**. Blue indicates amplification and red deletion. A closer examination of the figure demonstrates that for each patient a *different *non-empty set of genes is being targeted by genomic alterations, but the pathway is targeted in one form or another across the set.

**Figure 4:**
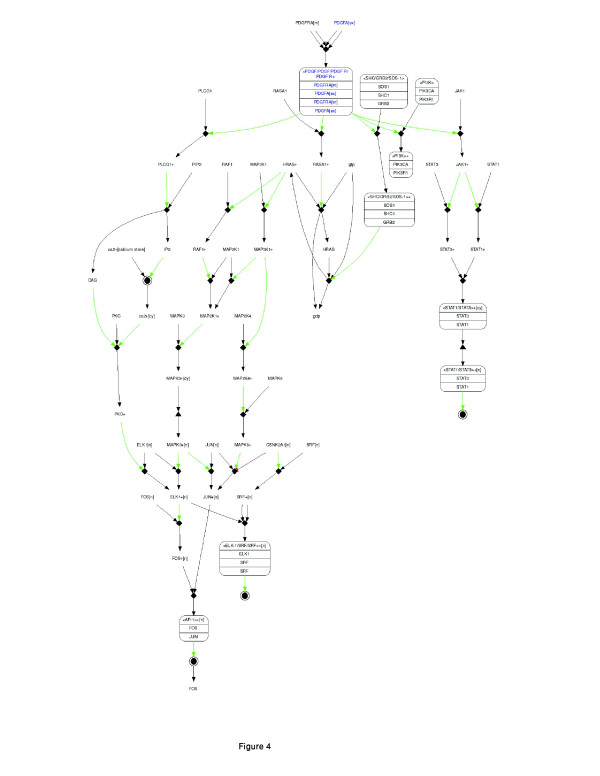
**The PDGF pathway diagram taken from NCI's Pathway-Interaction-Database (PID)**. Pathway members and the interactions between them are used as the basis for the computational metric of pathway behavior. Interactions are quantified according to gene-expression abundance and are iterated across the pathway.

The analysis revealed that higher levels of the PDGF pathway activity are associated with lower survival rates. Figure 2 gives KM curves, based on the pathway's activity, across the data sets. To study the molecular characteristics of this pathway further, we made use of the intensive molecular features available through TCGA. We analyzed the copy number and methylation profiles of the pathway genes. We took an approach that statistically quantifies the bias within the set of genes, according to their genomic modifications. This approach is detailed in [29]. The method uses Fisher's omnibus [30,31] to assign a p-value to each sub-network according to genomic events (such as copy number variation). Pathways with a gene set enriched with genomic events (across all 511 patients) are assigned lower p-values. Using this metric, we found the PDGF pathway provides highly significant p-values (P-value: 0.01) when considered from a copy number alterations perspective. Figure 3 shows changes in copy number across genes in this pathway; blue indicates amplification and red deletion. The figure demonstrates that practically every patient in the study had undergone change in copy number in multiple genes in the pathway. The use in this statistical test enables us to quantify the genomic changes and to distinguish between changes that their occurrences are above normality. To account for the specific behavior of the gene content, we briefly discuss their specific behavior in relevance to current findings. Figure 4 outlines the pathway's gene content and the interrelations between genes according to the PID [28] database: JAKs (Janus kinases) are a family of tyrosine kinases associated with cytokine receptors. Upon receptor activation, JAKs phosphorylate transcription factors known as STATs and initiate the JAK-STAT signaling pathway. Activation of this pathway has been implicated in the pathogenesis of a variety of human malignancies; this activation promotes acceleration of cell proliferation, up-regulation of survival factors, and activation of antiapoptotic proteins [32,33]. ERK1 (extracellular signal regulated protein kinase) mediates key events throughout the cell. Recent studies have shown that persistent activation of ERK plays a major role in cell migration and tumor progression [34,35]. JUN is the putative transforming gene of avian sarcoma virus 17 and is a well-known proto-oncogene (when highly expressed it becomes oncogenic). It is central to cellular signal transduction and regulation of proliferation [36,37].

Careful examination of copy number alterations in the PDGF pathway reveals interesting behavior. These specific genes demonstrate combined differential behavior in the survival groups, as defined by the pathway's activity measure. Group1 (better survival and lower pathway activity) contains frequent deletion of genes. This group (group1) showed 17%, 4% and 20% deletion in JUN, ERK1 and JAK1 respectively; in contrast, group2 (lower survival rates and higher pathway activity) showed only 11%, 1% and 11% deletion percentiles respectively.

The deleted genes in this set are considered oncogenes and thus support tumor progression. Their deletion is consistent with the observed differences in survival rates. In addition, analysis of the correspondence between gene expression, copy number variation and methylation profile revealed differences between the groups in three genes in the pathway. JUN, a proto-oncogene, showed significant correlation (p < 0.05) between CNV and gene-expression levels in both groups, but there was also a significant positive correlation (p = 0.0145) between methylation and copy number levels in Group1 (better survival); this correlation was absent in Group2 (poor prognosis). This positive correlation indicates that when Group1 gains more copies of JUN it is also has higher levels of methylation. This may indicate a mechanism that compensates the amplifications in JUN by silencing JUN with methylation. This mechanism can only be seen in the better survival group, once again consistent with the differences in survival rates. Two more genes that showed differences in the triple profile between the two groups are PLCG1 and STAT3. Both are involved in intracellular signaling cascades and are known to be involved in tumorigenesis, proliferation and cell survival [38-41]. PLCG1 and STAT3 showed significant positive correlations between CNV and gene-expression levels in both groups, but there were also significant negative correlations (PLCG1 p-value = 0.035, STAT3 p-value = 0.033) between the methylation and gene-expression levels in Group2 (poor prognosis) and not in Group1 (better survival). When those genes are amplified, the methylation levels are low, meaning that the patients concerned had gained active copies that were not silenced by methylation. Furthermore, examination of gene-expression levels in Group1 demonstrates a strong positive correlation between the expression levels of Jun, a well-known proto-oncogene, and FOS (Additional file 3 Table S3).

The PDGF signaling pathway has been extensively studied and well characterized since PDGF was first described in the 1970's as a serum factor that promoted the smooth muscle cell proliferation [42]. PDGF receptors are expressed in 50%-70% of ovarian tumors, recent studies on the PDGF signaling pathway in ovarian cancer suggests an over expression of the pathway due to over expression in the PDGF receptor which initiate the entire pathway. Thus, lead to the assumption that inactivation of the PDGF signaling by novel approaches is likely to have a significant impact in cancer therapy [43-45]. The increased evidences to the over-expression of the PDGF signaling together with its important role in almost all aspects of cancer biology, including migration, apoptosis, angiogenesis and metastasis joins and strengthen the results shown here and emphasizes the importance of the PDGF signaling pathway in ovarian cancer progression.

Ovarian cancer survival rates vary dramatically with stage. Within any stage, however, differences are noted in survival by age: younger women have better prognoses than older women, even after adjustment for the general life expectancy of each age group (relative survival) [46]. Moreover, among patients with suboptimal (> 1 cm residual disease) epithelial ovarian cancer, those who have small diameter residual disease (< 2 cm) tend to survive longer than those who have larger residual disease. Among those with larger residual disease, diameter does not affect prognosis appreciably [47].

**Figure 5:**
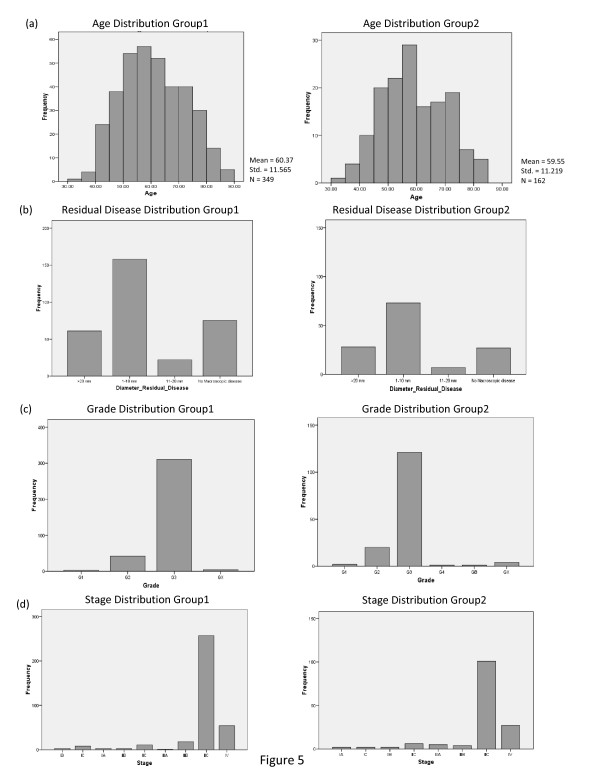
**Distribution of clinical features in the two groups stratified by the PDGF metrics: (A) Age, (B) Diameter of residual disease, (C) Stage, (D) and Grade distribution between the two groups**. The figure demonstrates that the two groups display very similar clinical features.

To confirm that stratification into groups as performed here through pathway analysis is indeed based solely on the metric and is not a recapitulation of clinical variables, we performed additional analysis on the correlation between the clinical measurements assessed and the groups that emerged. This analysis revealed that the classification was indeed a consequence of pathway activity and not a recapture of well-known clinical features, demographic features or disease history. Figure 5 shows clinical measurement distributions in the two groups.

## Conclusions

Over the past few decades, different genes have been used, with greater or lesser success, as biomarkers for prognostics. In the work presented here, by performing genome-wide sequential analyses across all genes and across all pathways, starting with TCGA and validating in two additional datasets, we saw how the single-gene approach fails to stratify patients robustly into prognostic groups. By applying the same strategy but with a different metric, that of pathway modifications, we identified one pathway that significantly and consistently stratified prognosis across the TCGA set and the two additional validation sets. In marked contrast, the expression levels of the genes composing the pathway did not provide valid prognosis stratification.

Methylation, copy-number variation and gene-expression have been established as molecular markers of tumor formation. Here, by looking into these genetic and epigenetic modifications in the PDGF pathway, we found this pathway to be significantly targeted by changes in copy number. Alterations in copy numbers may provide a causal explanation of why this pathway is a valid classifier. The expression level of a gene, in and of itself, fails to produce similar results; it is only the combined, synthetic, synergistic effects of subnetworks that identify phenotype affiliation. By isolating specific subnetworks, we were able to handle the NP-hard numeracy of network interactions. Further analysis revealed specific interactions at the core of the phenotypic clustering.

The lack in robustness of a single gene or even a set of genes emphasizes the importance of the pathway structure. While in a gene-set analysis every gene has the same weight of importance, in a pathway analysis a gene in calculated according to its location and contribution to the pathway.

Interestingly, expression levels of FOS are often higher in patients with a good prognosis than patients with poor prognosis. Studies on the oncogenic functions of FOS show it to be involved in the regulation of tumorogenesis, leading to down-regulation of tumor suppressor genes and eventually to invasive growth of cancer cells [48,49]. In contrast, other studies have shown FOS to act as a tumor suppressor gene. The authors of a recent study on epithelial ovarian carcinoma showed that reduction in FOS expression was associated with significantly shorter overall survival rates. They explained that the tumor-suppressor activity of FOS could be a pro-apoptotic function, which might confer increased chemoresistance on tumors with low FOS protein levels [50].

This JUN-FOS correlation was robustly present in Group1 throughout the three datasets, but there was no similar JUN-FOS correlation in Group2. This consistent correlation in the better survival group and the consistent lack of correlation in the second group lead us to propose that the prognosis-related correlation is highly significant and may indeed account for the differences in survival. A positive correlation indicates similar intracellular behavior: when JUN expression levels are high, FOS expression levels are high (and vice versa). That is, in well-controlled cases (better prognosis), when JUN behaves as an oncogene (high expression levels), FOS is highly expressed to suppress and oppose JUN activity. This behavior disappears in the poor prognosis cases, where this control mechanism fails and the gene correlation falls. Owing to their known close connection [51,52] and their opposite functions in tumorigenesis, we assumed that the correlation in the better survival group and the lack of correlation in the poor prognosis group are not coincidental and are strongly connected to the prognostic outcome. In addition, the fact that neither FOS nor JUN alone stratified prognosis consistently across the three datasets supports the assumption that only their co-behavior in the PDGF pathway can potentially be a target for future therapeutics.

Our results demonstrate that pathway interactions are either associated with improved prognosis by "helping" the pathway counter the tumor, or with poor prognosis by "breaking down" the pathway's normal activity. Through better understanding of the pathway mechanisms and the interactions that undergo changes, we may find targets for new treatments. The fact that the pathway we identified did not correlate with age or tumor diameter and was found in all three datasets strengthens the hypothesis that this pathway is a core mechanism of the disease.

Recent study on the ovarian cancer dataset from the TCGA found a 193-gene signature that predict overall survival in the TCGA data and additional datasets [53]. Interestingly, the pathway presented here outperforms the 193-gene signature in both the kaplan-meier p-value in the TCGA database (p-value of 0.02 compare to 0.007 in our results) and the number of genes in the prognosis classification (193 gene compare to 18 genes in the PDGF pathway). The work presented here, along with other studies, emphasizes the network unit as a biomarker [54,55]. By making the transition from the gene as the unit of phenotypic affiliation to the molecular network as the unit of analysis, we obtained highly significant prognosis curves. Furthermore, this transition to the process instead of the single agent facilitates the discovery of a process-based classification.

## Methods

### Gene Datasets

#### 1. TCGA

All data were obtained from The Cancer Genome Atlas (TCGA) database, available at http://cancergenome.nih.gov/. This dataset comprises molecular characterizations from over 500 ovarian cancer patients. For each patient, the database provides methylation, copy number and microarray values. In addition, the following clinical data variables were recorded for each patient: age, tumor grade, tumor stage, vital status and tumor histology. DNA methylation levels were quantified using an Illumina Infinium HumanMethylation27 BeadChip, which quantifies 27,578 highly informative CpG sites located within the proximal promoter region of transcription start sites of 14,475 consensus coding sequences (http://www.illumina.com/products/infinium_humanmethylation27_beadchip_kits.ilmn). The BeadChip technology allows as little as 2.5% methylation to be detected at a specific site. Furthermore, it can distinguish 17% differences in absolute methylation level between samples. [56]. The methylation status of an interrogated CpG site is determined by calculating the beta value, defined as the ratio of the fluorescence signal from the methylated allele to the sum of the fluorescence signals of both methylated and unmethylated alleles [10]. The beta value is between 0 and 1, where 0 is fully unmethylated and 1 is fully methylated. In our analyses, values over 0.5 were tagged methylated and values below 0.5 were tagged unmethylated. CNV levels obtained from the Human Genome CGH 244A microarray. [57]. CGH arrays provide a means for quantitative measurement of DNA copy number aberrations and for mapping them directly on to genome sequences. A value of 0 (log 2 ratio) indicates a normal state, 1 indicates 2 copy gains and -1 refers to heterozygous deletion. A standard threshold for copy number alteration of >0.3 for amplification and < -0.3 for deletion was applied as previously described by [58-60]. Gene-expression was quantified using an Affymetrix HT Human Genome U133 Array Plate Set. The expression data were normalized by quintile normalization to produce RMA expression values from the Affymetrix CEL files. All CEL files from all batches have been normalized together to produce RMA expression values. This has been done with the purpose of avoiding technical variation such as batch effect. RMA has been extensively used in such studies and had become the de facto standard in normalizing Affymetrix mRNA expression data. Further, to reduce any biases we performed two additional validations in additional data sets. TCGA consortium have recently published a comprehensive work on ovarian cancer data [53] in which they demonstrate a substantial batch effect across Agilent and Affymetrix Human Exon Arrays, which suffer from sever batch effects. Yet, the Affymetrix U133A platform showed only modest batch effects.

Gene expression in all three datasets was analyzed on the normalized RMA expression data.

#### 2. Duke university medical center dataset #1

The dataset is composed of gene-expression and clinical information from 119 patients. All ovarian cancer samples were obtained at initial cytoreductive surgery from patients treated at Duke University Medical Center and the H. Lee Moffitt Cancer Center and Research Institute, who then received platinum-based primary chemotherapy [15]. Gene-expression was quantified using the Affymetrix Human Genome U133A Array.

#### 3. Duke university medical center dataset #2

The dataset is composed of gene-expression and clinical information from 42 patients. All ovarian cancers samples collected from the primary ovarian site were snap-frozen at initial surgery prior to chemotherapy under the auspices of Institutional Review Board-approved tissue collection protocols [14]. Gene-expression was quantified using the Affymetrix Human Genome U133A Array.

### Pathway network interactions dataset

Network information was obtained from the National Cancer Institute's Pathway Interaction Database [28].

#### Gene-Expression analysis

Pathway Consistency and Pathway Activity metrics were calculated according to [26] and [27,61]. These measures treat the pathway as a network of interactions and give the network a score based on the expression levels of each of the genes in the interaction and on the quality of the interaction. The analysis takes into consideration the specific type of interaction (such as inhibition or promotion).

The Activity is a measure of the likelihood that the interaction occurs in the pathway. When taking a pathway with two genes as input and one gene as output, the algorithm calculates their probability of being in an "up" state (by taking into account the expression levels of those genes in all the samples). The activity of this pathway is the probability that this interaction is "active", meaning the product of the probabilities that the two genes are in the "up" state. The Consistency is a measure comparing the expected vs. actual expression of the interaction components, obtained by calculating the probabilities of an (i) active interaction, (ii) that the output gene is in an "up" state, and (iii) of the complementary event.

The probability of a gene to be either "up" or "down" is calculated using its expression value (RMA adjusted) in a sample, compared to the expression values of the same gene in all other samples. To be able to accommodate a multitude of probability distributions, the algorithm uses a gamma distribution as the template to both "down" and "up" distributions form, and redefines the problem as a mixture of two gamma distribution. The suppressed form often follows an exponential distribution, which is one particular case of a gamma distribution. The promoted state often follows a form similar to a normal distribution, which may be approximated by a gamma distribution of a large mean. Per every probe set measured by the microarray, the algorithm fit the expression distribution into a mixture of two gamma distributions. Additional file 4 Figure 1 describes the algorithm.

genomic pathways targeting analysis Targeting of pathways by genomic and epigenomic alterations was calculated according to [29]. Fisher's omnibus test is a well known test for detecting deviations from normality due either to skewness or kurtosis [30]. Applying this statistical test in pathway analysis is a way of determining whether the CNV and methylation status of a pathway is above normal. Applying this analysis on large-scale data enables researchers to extract pathways with significant alterations in CNV and methylation status. All genes in both copy number and methylation datasets were matched to their corresponding pathways. The probability for the pathway alteration in every subject was calculated using hyper-geometric function as follow:

$$\begin{array}{c} X_{i}-The\;number\;of\;altered\;genes\;in\;a\;given\;patient\;in\;pathway\;i\\ K_{j}-Number\;of\;altered\;genes\;in\;patient\;j\\ M-Total\;number\;of\;genes\;tested\\ N_{i}-Number\;of\;genes\;in\;pathway\;i\\ p=F\left(x|M,K,N\right)=1-\overset{x}{\underset{i=0}{\sum }}\frac{\left(\begin{array}{c} K\\ i \end{array}\right)\left(\begin{array}{c} M-K\\ N-i \end{array}\right)}{\left(\begin{array}{c} M\\ N \end{array}\right)} \end{array}$$

The result is the probability of hitting up to x of possible K genes in N drawings. P-value for every pathway was then calculated using Fisher's omnibus test to establish pathways that go through significant targeting by genomic and epigenomic alterations. Bonferroni correction for multiple hypotheses was applied and pathways with p-value under 8.6356 × 10^-5 ^(0.05/579 pathways in analysis) were then chosen as highly targeted.

#### Survival analysis

Kaplan-Meier survival analysis was done on all gene and pathway measurements in all three datasets [62], through clinical data (Vital Status) to determine a pathway's and gene's survival stratification power. This analysis was done in order to find genes or pathways that could stratify prognosis in all three datasets.

All values (RMA of gene, and pathway activity and consistency) were clustered using K-means clustering to stratify the patients into two groups according to their expression values (meaning patients with lower expression values clustered into group1 and patients with high expression values clustered into group2). Kaplan-Meier (KM) analysis was then performed on all genes and pathways matrix (according to the k-means group and the vital status). Genes and pathways that had significant Kaplan-Meier p values (< 0.05) were then chosen as good separators for prognosis. All the results were then compared in all three datasets in order to identify overlapping genes and pathways.

## Competing interests

The authors declare that they have no competing interests.

## Authors' contributions

RBH and SE designed analyzed and wrote the paper. All authors read and approved the final manuscript.

## Supplementary Material

Additional file 1 **Genes Kaplan-Meier p-value**. The table presents the gene symbol, gene probe and kaplan-meier log-rank p-value of all the significant genes in the three datasets. Click here for file

Additional file 2 **Pathways Kaplan-Meier p-value**. The table presents the pathways name and kaplan-meier log-rank p-value of all the significant pathways in the three datasets. Click here for file

Additional file 3 **FOS-JUN Correlation**. The table presents the correlations between FOS and JUN, which are eventually the pathways output, in the two survival group. Group1, which correlates with higher survival rates, shows stronger correlation between the genes. Click here for file

Additional file 4 **Pathway calculation algorithm pipeline**. The figure describes the calculation steps performed by the PathOlogist algorithm, starting with the RMA gene-expression levels. Click here for file
