# Supplementary material for: Biomarker robustness reveals the PDGF network as driving disease outcome in ovarian cancer patients in multiple studies
Source: BMC Syst Biol. 2012 Jan 11;6:3. doi: 10.1186/1752-0509-6-3 (PMC3298526; doi:10.1186/1752-0509-6-3)
Supplement: Additional file 1 — Genes Kaplan-Meier p-value. The table presents the gene symbol, gene probe and kaplan-meier log-rank p-value of all the significant genes in the three datasets. [file 1752-0509-6-3-S1.PDF]

| TCGA (511 Patients) | | | Duke (119 Patients) | | | Duke (42 Patients) | | |
|---|---|---|---|---|---|---|---|---|
| Gene Symbol | Probesets | Logrank - Pval | Gene Symbol | Probe | Logrank - Pval | Gene Symbol | Probe | Logrank - Pval |
| GMNN | 218350_s_at | 0.042822 | PRKCSH | 200707_at | 0.041753 | FYN | 216033_s_at | 0.033893 |
| MYCBP | 203359_s_at | 0.022819 | SSR1 | 200890_s_at | 0.037056 | FOXO1 | 202723_s_at | 0.033006 |
| PIP5K1C | 212518_at | 0.033239 | RPLP2 | 200908_s_at | 0.031215 | IKBKB | 209341_s_at | 0.017142 |
| NUP153 | 202097_at | 0.042822 | RBBP7 | 201092_at | 0.043817 | IKBKB | 209342_s_at | 0.02596 |
| NXT2 | 209628_at | 0.001069 | FAM32A | 201863_at | 0.043662 | IKBKB | 211027_s_at | 0.03987 |
| STRA13 | 209478_at | 0.004277 | NUP153 | 202097_at | 0.035661 | MAPT | 203929_s_at | 0.02596 |
| GCH1 | 204224_s_at | 0.004606 | FARSA | 202159_at | 0.046644 | FGFR1 | 215404_x_at | 0.034808 |
| KYNU | 217388_s_at | 0.005287 | PIK3R3 | 202743_at | 0.041051 | MAP2K5 | 216435_at | 0.050016 |
| CXCL11 | 211122_s_at | 0.005975 | CREBZF | 202979_s_at | 0.036795 | VEGFA | 210513_s_at | 0.026552 |
| WDR46 | 209196_at | 0.006458 | KRR1 | 203202_at | 0.018412 | AMT | 204294_at | 0.03987 |
| YIPF6 | 212340_at | 0.006602 | MYCBP | 203359_s_at | 0.021323 | VEGFA | 211527_x_at | 0.043114 |
| RPL12 | 214271_x_at | 0.007 | PNPLA6 | 203718_at | 0.045187 | NF1 | 216115_at | 0.008109 |
| IL2RG | 204116_at | 0.007482 | ACTA1 | 203872_at | 0.039679 | IRF4 | 216987_at | 0.026091 |
| IRF4 | 204562_at | 0.007683 | PMAIP1 | 204285_s_at | 0.027165 | IGFBP1 | 205302_at | 0.03987 |
| LSAMP | 214460_at | 0.007776 | CNKSR1 | 204740_at | 0.037151 | POLR2A | 217415_at | 0.050016 |
| CCL7 | 208075_s_at | 0.007899 | TMEM5 | 204808_s_at | 0.048061 | GTSE1 | 211040_x_at | 0.005088 |
| SETD4 | 213989_x_at | 0.008349 | CLTB | 205172_x_at | 0.049686 | SMAD1 | 208015_at | 0.016382 |
| HCP5 | 206082_at | 0.008616 | SMAD5 | 205188_s_at | 0.042131 | MARCKS | 213002_at | 0.019349 |
| DAXX | 216038_x_at | 0.009046 | PAFAH2 | 205232_s_at | 0.007254 | DBT | 205370_x_at | 0.019402 |
| TRAC | 209670_at | 0.009124 | HNF1B | 205313_at | 0.044469 | TCF3 | 213731_s_at | 0.02596 |
| ETF1 | 201573_s_at | 0.011203 | RCE1 | 205333_s_at | 0.041753 | --- | 220882_at | 0.050016 |
| ASAP3 | 219103_at | 0.01206 | TBKBP1 | 205424_at | 0.022359 | INADL | 214705_at | 0.026091 |
| CXCL11 | 210163_at | 0.012077 | FOXO4 | 205451_at | 0.039679 | TNFRSF4 | 208023_at | 0.026888 |
| WDR25 | 219609_at | 0.012719 | HSPB2 | 205824_at | 0.02446 | CD46 | 208783_s_at | 0.030681 |
| NR1H3 | 203920_at | 0.012956 | CLTCL1 | 205944_s_at | 0.02476 | SPN | 206056_x_at | 0.033138 |
| MICB | 206247_at | 0.013119 | OPCML | 206215_at | 0.022697 | PGK1 | 217356_s_at | 0.033138 |
| ISG20 | 33304_at | 0.013799 | PTAFR | 206278_at | 0.02446 | CTTN | 214782_at | 0.034808 |
| IGJ | 212592_at | 0.013893 | --- | 206863_x_at | 0.040326 | GTSE1 | 204315_s_at | 0.038607 |
| SREBF2 | 201247_at | 0.014007 | PDIA2 | 206889_at | 0.021847 | PERP | 217744_s_at | 0.038607 |
| CXCL9 | 203915_at | 0.014486 | ZNF141 | 206931_at | 0.028424 | SH2D2A | 207351_s_at | 0.042572 |
| CD44 | 212014_x_at | 0.014534 | BRS3 | 207369_at | 0.039667 | CASP4 | 213596_at | 0.042572 |
| SNIP1 | 219409_at | 0.014654 | EXOSC10 | 207541_s_at | 0.037151 | FSHB | 214489_at | 0.043114 |
| PDCD10 | 210907_s_at | 0.014683 | GRK1 | 208041_at | 0.049634 | KLRD1 | 207796_x_at | 0.050016 |
| TAF15 | 202840_at | 0.015163 | TMX1 | 208097_s_at | 0.048061 | N4BP2L2 | 202258_s_at | 0.002569 |
| PPP3CA | 202425_x_at | 0.015256 | CACNA1E | 208432_s_at | 0.043931 | PDCD6 | 222152_at | 0.002901 |
| CD3D | 213539_at | 0.015312 | CLCN1 | 208437_at | 0.045187 | SRP72 | 208803_s_at | 0.004639 |
| DOM3Z | 215982_s_at | 0.015575 | CD164 | 208653_s_at | 0.045024 | LOC100272216 | 213089_at | 0.004639 |
| TRA@ /// TRAC | 209671_x_at | 0.01586 | CDC42 | 208728_s_at | 0.009341 | CTTNBP2NL | 214731_at | 0.004639 |
| RAB3GAP1 | 213531_s_at | 0.015876 | GORASP2 | 208843_s_at | 0.013941 | MAPK1IP1L | 212497_at | 0.005088 |
| CCL5 | 1405_i_at | 0.016339 | NCOR2 | 208889_s_at | 0.031481 | --- | 217446_x_at | 0.005088 |
| RPAP2 | 219504_s_at | 0.016339 | KPNB1 | 208975_s_at | 0.025185 | BBX | 213016_at | 0.005354 |
| TRBC1 | 213193_x_at | 0.016625 | TRIP6 | 209129_at | 0.010459 | SLC35E1 | 220796_x_at | 0.005613 |
| MED20 | 212872_s_at | 0.016762 | KLHDC10 | 209254_at | 0.041051 | --- | 216858_x_at | 0.005699 |
| SERPINA2 | 208531_at | 0.016797 | PRKAA1 | 209799_at | 0.037056 | PRR11 | 219392_x_at | 0.005699 |
| TRPV2 | 219282_s_at | 0.017627 | RBMS1 | 209868_s_at | 0.033199 | CCNT2 | 204645_at | 0.008109 |
| ATP2B1 | 209281_s_at | 0.018104 | PCDH7 | 210273_at | 0.026582 | GTF2H2B | 215470_at | 0.008109 |
| CTNND2 | 209618_at | 0.018123 | ZMYM2 | 210282_at | 0.037991 | --- | 217679_x_at | 0.008109 |
| CDC14B | 208022_s_at | 0.018138 | B4GALT4 | 210540_s_at | 0.044537 | LPIN1 | 212274_at | 0.009584 |
| ARL4C | 202206_at | 0.018393 | SPPL2B | 210693_at | 0.013294 | CDV3 | 213548_s_at | 0.009584 |
| MRS2 | 218538_s_at | 0.01849 | CCKAR | 211173_at | 0.049634 | LOC152719 | 215978_x_at | 0.009584 |
| RPL22 | 221726_at | 0.018531 | PTGER3 | 211909_x_at | 0.021847 | AGER | 217046_s_at | 0.009584 |
| ISG20 | 204698_at | 0.018622 | CFB | 211920_at | 0.034293 | HNRNPL | 221860_at | 0.009584 |

| | | | | | | | | |
|---|---|---|---|---|---|---|---|---|
| CCDC90A | 220094_s_at | 0.01883 | KIAA0090 | 212395_s_at | 0.045024 | AGGF1 | 208042_at | 0.010325 |
| TAP1 | 202307_s_at | 0.018891 | KIAA0232 | 212441_at | 0.045024 | SPIN1 | 217813_s_at | 0.010515 |
| RPL12 | 200809_x_at | 0.019151 | CARM1 | 212512_s_at | 0.013306 | ZNF767 | 219627_at | 0.010782 |
| PPP3CA | 202457_s_at | 0.019151 | PIP5K1C | 212518_at | 0.028303 | POLR1B | 220113_x_at | 0.010782 |
| SLA | 203761_at | 0.019774 | CTSZ | 212562_s_at | 0.035661 | --- | 215398_at | 0.011085 |
| NCF1C | 214084_x_at | 0.019787 | STX10 | 212625_at | 0.033199 | MSH3 | 205887_x_at | 0.012612 |
| CCR7 | 206337_at | 0.019904 | CAMSAP1L1 | 212765_at | 0.026251 | MYL12A | 201319_at | 0.013628 |
| ADAMDEC1 | 206134_at | 0.020056 | POLD3 | 212836_at | 0.039056 | PLOD2 | 202619_s_at | 0.013628 |
| CCHCR1 | 37425_g_at | 0.020056 | BBX | 213016_at | 0.045187 | USP34 | 207365_x_at | 0.013628 |
| MMP9 | 203936_s_at | 0.020194 | SH3BP1 | 213633_at | 0.039667 | ZNF611 | 208137_x_at | 0.013628 |
| CDC14B | 221555_x_at | 0.020707 | --- | 213642_at | 0.027243 | --- | 214977_at | 0.013628 |
| IGHG1 | 217260_x_at | 0.020742 | TMEM110 | 213851_at | 0.017679 | --- | 215387_x_at | 0.013628 |
| PYCR1 | 202148_s_at | 0.021463 | TWF1 | 214007_s_at | 0.024028 | --- | 220728_at | 0.013628 |
| ATP5J2 | 202961_s_at | 0.021561 | MAGEA4 | 214254_at | 0.023435 | --- | 217662_x_at | 0.015861 |
| ATP2B1 | 212930_at | 0.021773 | MTMR1 | 214975_s_at | 0.047887 | IGF2R | 201392_s_at | 0.016382 |
| MARS | 201475_x_at | 0.022051 | PICALM | 215236_s_at | 0.047887 | CALM1 | 213688_at | 0.016382 |
| SLAMF8 | 219385_at | 0.022547 | TNPO2 | 215844_at | 0.027011 | AKAP11 | 215336_at | 0.016382 |
| MMP19 | 204574_s_at | 0.022586 | --- | 216101_at | 0.038039 | --- | 215609_at | 0.016382 |
| SDF2L1 | 218681_s_at | 0.02299 | --- | 216246_at | 0.040326 | --- | 216751_at | 0.016382 |
| PLA2G2D | 220423_at | 0.023392 | --- | 216410_at | 0.028009 | --- | 217701_x_at | 0.016382 |
| TNNT2 | 215389_s_at | 0.023932 | DUX4 | 216473_x_at | 0.02446 | MECOM | 208434_at | 0.016678 |
| ACHE | 205377_s_at | 0.023979 | --- | 216586_at | 0.041051 | --- | 215344_at | 0.016678 |
| RALGPS1 | 204199_at | 0.024256 | RBM | 216842_x_at | 0.045187 | --- | 216729_at | 0.016678 |
| CASK | 211208_s_at | 0.024405 | SMOX | 217074_at | 0.021438 | NSUN5B | 214100_x_at | 0.017142 |
| PSMB2 | 200039_s_at | 0.024968 | --- | 217438_at | 0.027547 | SCIN | 222272_x_at | 0.017142 |
| HLA-C | 211799_x_at | 0.025848 | SMC1A | 217555_at | 0.021111 | SPG21 | 215383_x_at | 0.018158 |
| TNFRSF4 | 214228_x_at | 0.026156 | C19orf56 | 217780_at | 0.028009 | CNOT2 | 222182_s_at | 0.018158 |
| TRAK1 | 214924_s_at | 0.026189 | SYNCRIP | 217834_s_at | 0.026582 | MGEA5 | 200899_s_at | 0.018372 |
| EGR1 | 201693_s_at | 0.026416 | ZDHHC3 | 218077_s_at | 0.01755 | VAPA | 208780_x_at | 0.018372 |
| ZFHX4 | 219779_at | 0.026546 | ISOC1 | 218170_at | 0.035634 | BCAT2 | 215654_at | 0.018372 |
| SNRPC | 201342_at | 0.027471 | GMNN | 218350_s_at | 0.018466 | SETD4 | 219482_at | 0.018372 |
| PLEKHA5 | 220952_s_at | 0.027471 | HAUS4 | 218383_at | 0.037056 | N4BP2L2 | 221899_at | 0.018372 |
| APC | 216933_x_at | 0.027521 | FANCL | 218397_at | 0.048061 | --- | 222358_x_at | 0.018372 |
| SVIL | 202565_s_at | 0.02757 | DHDDS | 218547_at | 0.020372 | TM9SF1 | 209149_s_at | 0.019349 |
| CXCL10 | 204533_at | 0.027593 | RRP1 | 218758_s_at | 0.034308 | PCNT | 203660_s_at | 0.019402 |
| LOC100293324 | 216510_x_at | 0.027687 | KRI1 | 218798_at | 0.041753 | KIAA0894 | 207436_x_at | 0.019402 |
| LYZ | 213975_s_at | 0.027721 | GPATCH3 | 218895_at | 0.026346 | BICD2 | 209203_s_at | 0.019402 |
| MAPK13 | 210058_at | 0.027792 | TTC17 | 218972_at | 0.045187 | FBXW12 | 215600_x_at | 0.019402 |
| EPHB3 | 1438_at | 0.027946 | PGBD5 | 219225_at | 0.048936 | PUS1 | 218670_at | 0.019402 |
| EEF1E1 | 213907_at | 0.027946 | EGLN3 | 219232_s_at | 0.038039 | UTP20 | 209725_at | 0.020938 |
| TARP /// TRGC2 | 215806_x_at | 0.02809 | PHACTR4 | 219235_s_at | 0.026346 | SMN1 | 203852_s_at | 0.022119 |
| EGR3 | 206115_at | 0.028124 | ESPN | 219422_at | 0.040326 | PDCL | 204448_s_at | 0.022119 |
| C6orf62 | 222309_at | 0.028157 | PLA1A | 219584_at | 0.033687 | --- | 206548_at | 0.022119 |
| RPL12 | 200088_x_at | 0.02834 | BANP | 219966_x_at | 0.039667 | MBTD1 | 207115_x_at | 0.022119 |
| FAT1 | 201579_at | 0.028403 | FAR2 | 220615_s_at | 0.033687 | ALPK1 | 207133_x_at | 0.022119 |
| TRAV20 | 210972_x_at | 0.028403 | --- | 220969_s_at | 0.040251 | NDUFB8 | 214241_at | 0.022119 |
| ACTL8 | 214957_at | 0.028502 | GTPBP8 | 221046_s_at | 0.044469 | PLGLB1 | 214415_at | 0.022119 |
| MAPK13 | 210059_s_at | 0.028548 | TNPO2 | 221507_at | 0.046644 | DIP2A | 215529_x_at | 0.022119 |
| GAS1 | 204456_s_at | 0.028598 | LRCH4 | 222017_x_at | 0.044469 | --- | 217659_at | 0.022119 |
| LOC646057 | 217384_x_at | 0.028654 | SIRT5 | 222080_s_at | 0.043662 | TMEM33 | 218465_at | 0.022119 |
| SLAMF7 | 219159_s_at | 0.029 | ZNF142 | 37586_at | 0.041051 | TAF1D | 218750_at | 0.022119 |
| ITGB1 | 215879_at | 0.029046 | AP1M2 | 65517_at | 0.028009 | ABHD2 | 205566_at | 0.022895 |
| ADD1 | 214736_s_at | 0.029312 | ABHD2 | 87100_at | 0.030208 | POFUT1 | 210433_at | 0.02596 |
| CD48 | 204118_at | 0.029477 | | | | --- | 215810_x_at | 0.02596 |
| PLAA | 209533_s_at | 0.029616 | | | | CES3 | 220335_x_at | 0.02596 |
| CLSTN1 | 201561_s_at | 0.030083 | | | | SMARCC2 | 201321_s_at | 0.026091 |

| | | | | | | | | |
|---|---|---|---|---|---|---|---|---|
| CCDC41 | 219644_at | 0.030327 | | | | PCNXL2 | 205689_at | 0.026091 |
| ITGA3 | 201474_s_at | 0.030363 | | | | WDR1 | 210935_s_at | 0.026091 |
| GLO1 | 200681_at | 0.030862 | | | | LOC150759 | 213703_at | 0.026091 |
| GTF2E1 | 205930_at | 0.030862 | | | | ACSM3 | 205942_s_at | 0.026552 |
| UQCRB | 209065_at | 0.030862 | | | | API5 | 214959_s_at | 0.026552 |
| UTP11L | 218235_s_at | 0.031428 | | | | SFRS8 | 202774_s_at | 0.026888 |
| NR2F1 | 209506_s_at | 0.031552 | | | | CNKSR1 | 204740_at | 0.026888 |
| ELN | 212670_at | 0.031552 | | | | PRDX2 | 215067_x_at | 0.026888 |
| ASB7 | 219996_at | 0.031676 | | | | PRKAG2 | 215231_at | 0.026888 |
| PTDSS2 | 221005_s_at | 0.031676 | | | | --- | 215828_at | 0.026888 |
| NAPG | 210048_at | 0.031751 | | | | --- | 216094_at | 0.026888 |
| WDFY3 | 212606_at | 0.031843 | | | | DGCR8 | 91617_at | 0.026888 |
| IFI35 | 209417_s_at | 0.031883 | | | | SNRNP70 | 201221_s_at | 0.030607 |
| MEN1 | 202645_s_at | 0.032145 | | | | ZBTB48 | 205025_at | 0.030607 |
| LIMD2 | 218600_at | 0.032318 | | | | PHF20 | 206567_s_at | 0.030607 |
| FGF21 | 221433_at | 0.032671 | | | | TTC3 | 210645_s_at | 0.030607 |
| ZCCHC14 | 215426_at | 0.032875 | | | | FAF2 | 212106_at | 0.030607 |
| UCP3 | 219827_at | 0.033412 | | | | --- | 215401_at | 0.030607 |
| ZNF322B | 219376_at | 0.033921 | | | | MAPKAP1 | 217808_s_at | 0.030607 |
| ATP2B1 | 215716_s_at | 0.034045 | | | | NDUFA8 | 218160_at | 0.030607 |
| PDE4B | 203708_at | 0.034102 | | | | FLJ42627 | 220352_x_at | 0.030607 |
| PIM2 | 204269_at | 0.034102 | | | | PCNXL2 | 39650_s_at | 0.030607 |
| HLA-DOB | 205671_s_at | 0.034313 | | | | PTP4A1 | 200730_s_at | 0.030681 |
| ZFP36 | 201531_at | 0.034463 | | | | DOLK | 204488_at | 0.030681 |
| GSPT1 | 215438_x_at | 0.034666 | | | | NRL | 206596_s_at | 0.030681 |
| GABBR1 /// UBD | 205890_s_at | 0.034701 | | | | PDE4C | 206792_x_at | 0.030681 |
| PPIH | 204228_at | 0.034857 | | | | AMPD3 | 207992_s_at | 0.030681 |
| FBXO9 | 212987_at | 0.035331 | | | | CAMSAP1L1 | 212763_at | 0.030681 |
| SPOCK2 | 202523_s_at | 0.035386 | | | | RIOK3 | 215588_x_at | 0.030681 |
| ABCF1 | 200045_at | 0.035472 | | | | CPSF3L | 217994_x_at | 0.030681 |
| CCNA2 | 203418_at | 0.035615 | | | | WDR60 | 219251_s_at | 0.030681 |
| INPP5F | 203607_at | 0.036417 | | | | ACTR2 | 200727_s_at | 0.033006 |
| FOS | 209189_at | 0.036417 | | | | PGRMC1 | 201120_s_at | 0.033006 |
| EDNRA | 204464_s_at | 0.036534 | | | | MARK2 | 203942_s_at | 0.033006 |
| JUN | 201466_s_at | 0.037151 | | | | KRIT1 | 204738_s_at | 0.033006 |
| HABP4 | 209818_s_at | 0.03736 | | | | MAFG | 204970_s_at | 0.033006 |
| NDUFB4 | 218226_s_at | 0.037528 | | | | --- | 208246_x_at | 0.033006 |
| RAD1 | 210216_x_at | 0.037544 | | | | ADAM7 | 211239_s_at | 0.033006 |
| CTSS | 202901_x_at | 0.037575 | | | | CLASP1 | 212752_at | 0.033006 |
| DAPK1 | 203139_at | 0.038161 | | | | LOC100272216 | 213605_s_at | 0.033006 |
| DOM3Z | 38157_at | 0.038161 | | | | MYO1C | 214656_x_at | 0.033006 |
| ITGA10 | 206766_at | 0.038201 | | | | --- | 215083_at | 0.033006 |
| ATP6V0B | 200078_s_at | 0.038257 | | | | --- | 215200_x_at | 0.033006 |
| DNASE2 | 214992_s_at | 0.038524 | | | | PARVA | 215418_at | 0.033006 |
| PLSCR1 | 202446_s_at | 0.038993 | | | | --- | 215774_s_at | 0.033006 |
| GABRA5 | 217280_x_at | 0.038993 | | | | XYLT2 | 219401_at | 0.033006 |
| TAF11 | 209358_at | 0.038999 | | | | ZNF133 | 37254_at | 0.033006 |
| GPR22 | 221288_at | 0.038999 | | | | TRAK2 | 202124_s_at | 0.033138 |
| ARL4C | 202208_s_at | 0.039021 | | | | SFRS8 | 202775_s_at | 0.033138 |
| AKAP12 | 210517_s_at | 0.039021 | | | | FAM115A | 204403_x_at | 0.033138 |
| CD3G | 206804_at | 0.039339 | | | | NVL | 207877_s_at | 0.033138 |
| CCR2 | 206978_at | 0.039339 | | | | ACOX1 | 209601_at | 0.033138 |
| ALG8 | 203545_at | 0.039421 | | | | KLK10 | 209792_s_at | 0.033138 |
| PHF20 | 209422_at | 0.039632 | | | | LRRFIP1 | 211452_x_at | 0.033138 |
| CD72 | 215925_s_at | 0.039764 | | | | --- | 215553_x_at | 0.033138 |

| | | | | | | | | |
|---|---|---|---|---|---|---|---|---|
| MANF | 202655_at | 0.039943 | | | | --- | 215628_x_at | 0.033138 |
| MBNL2 | 203640_at | 0.039943 | | | | TTC26 | 219758_at | 0.033138 |
| TRAF3IP3 | 213888_s_at | 0.040208 | | | | ZNF692 | 220661_s_at | 0.033138 |
| TNFSF13 | 211495_x_at | 0.04069 | | | | ZNF83 | 221645_s_at | 0.033138 |
| SERP1 | 200970_s_at | 0.040701 | | | | SEC62 | 208942_s_at | 0.033893 |
| STAT1 | AFFX-HUMISGF3A/M97935_3_at | 0.040701 | | | | ZBTB22 | 213081_at | 0.033893 |
| GZMB | 210164_at | 0.040757 | | | | SNRNP70 | 213121_at | 0.033893 |
| FEN1 | 204768_s_at | 0.040871 | | | | C6orf62 | 213872_at | 0.033893 |
| OR7A10 | 217316_at | 0.041102 | | | | SERPINB10 | 214539_at | 0.033893 |
| SH3BP2 | 209371_s_at | 0.041242 | | | | --- | 214912_at | 0.033893 |
| RGS14 | 204280_at | 0.041248 | | | | COMMD9 | 218072_at | 0.033893 |
| EXOSC4 | 91684_g_at | 0.041519 | | | | LRRC8D | 218684_at | 0.033893 |
| ZNF7 | 205089_at | 0.041806 | | | | KCNMB4 | 219287_at | 0.033893 |
| ZNF643 | 207219_at | 0.042108 | | | | DCLRE1C | 219678_x_at | 0.033893 |
| DEK | 200934_at | 0.042375 | | | | SRRT | 222047_s_at | 0.033893 |
| STK3 | 204068_at | 0.042375 | | | | BRD4 | 202102_s_at | 0.034808 |
| RANBP9 | 202582_s_at | 0.042837 | | | | POU2F1 | 206789_s_at | 0.034808 |
| TUBGCP4 | 213266_at | 0.042837 | | | | MED6 | 207078_at | 0.034808 |
| GPC1 | 202756_s_at | 0.042875 | | | | C7orf54 | 210109_at | 0.034808 |
| IGLV3-19 | 216853_x_at | 0.042907 | | | | MR1 | 210528_at | 0.034808 |
| ABCA1 | 216066_at | 0.042958 | | | | ASXL1 | 212238_at | 0.034808 |
| GJB1 | 204973_at | 0.042986 | | | | --- | 215576_at | 0.034808 |
| BTN2A2 | 205298_s_at | 0.043254 | | | | --- | 215586_at | 0.034808 |
| TIMP3 | 201148_s_at | 0.043533 | | | | NDRG3 | 217286_s_at | 0.034808 |
| SRGN | 201858_s_at | 0.043533 | | | | THUMPD2 | 219248_at | 0.034808 |
| DNASE2 | 209831_x_at | 0.043616 | | | | MLL | 220546_at | 0.034808 |
| ADAM10 | 202604_x_at | 0.043997 | | | | FAM128B | 220720_x_at | 0.034808 |
| PSMC3 | 201267_s_at | 0.044416 | | | | ACOXL | 220845_at | 0.034808 |
| LOC26102 | 216608_at | 0.044424 | | | | --- | 222282_at | 0.034808 |
| MGC29506 | 221286_s_at | 0.044461 | | | | CSPG4 | 204736_s_at | 0.038607 |
| CEP76 | 219311_at | 0.044595 | | | | LOC644213 | 222202_at | 0.038607 |
| IGL@ | 209138_x_at | 0.044872 | | | | PLA2R1 | 210194_at | 0.03987 |
| PPP1R15A | 37028_at | 0.044938 | | | | SETD1B | 213153_at | 0.03987 |
| HSPA13 | 202558_s_at | 0.044979 | | | | TUBB3 | 213476_x_at | 0.03987 |
| RND3 | 212724_at | 0.045042 | | | | ZNF573 | 217627_at | 0.03987 |
| SPG7 | 202104_s_at | 0.045146 | | | | MTRF1 | 219822_at | 0.03987 |
| C1orf109 | 218712_at | 0.045259 | | | | PMM1 | 203467_at | 0.042572 |
| FGF22 | 221315_s_at | 0.045352 | | | | WBP4 | 203597_s_at | 0.042572 |
| MTO1 | 218716_x_at | 0.045487 | | | | SH3PXD2A | 207661_s_at | 0.042572 |
| GPR56 | 206582_s_at | 0.045512 | | | | CNOT4 | 210204_s_at | 0.042572 |
| SNED1 | 213488_at | 0.045512 | | | | HSPA8 | 210338_s_at | 0.042572 |
| WARS | 200628_s_at | 0.045605 | | | | CDC2L5 | 210965_x_at | 0.042572 |
| STX7 | 212632_at | 0.045637 | | | | ZNF160 | 214715_x_at | 0.042572 |
| CUEDC1 | 219468_s_at | 0.045897 | | | | --- | 214989_x_at | 0.042572 |
| MSLN | 204885_s_at | 0.046014 | | | | PMS2L5 | 215410_at | 0.042572 |
| RALY | 201271_s_at | 0.046143 | | | | --- | 215587_x_at | 0.042572 |
| ACOT13 | 204565_at | 0.046143 | | | | HCRP1 | 216176_at | 0.042572 |
| PCDHAC2 | 210674_s_at | 0.046143 | | | | --- | 217164_at | 0.042572 |
| MRPL11 | 219162_s_at | 0.046839 | | | | RUFY2 | 219957_at | 0.042572 |
| FUT6 | 211465_x_at | 0.046959 | | | | --- | 220855_at | 0.042572 |
| LSM2 | 209449_at | 0.047262 | | | | CTNNBL1 | 221021_s_at | 0.042572 |
| IGK@ /// IGKC | 221651_x_at | 0.0474 | | | | TECTA | 221296_at | 0.042572 |
| MRPS18B | 217408_at | 0.04743 | | | | GDNF | 221359_at | 0.042572 |
| PHTF1 | 215285_s_at | 0.047454 | | | | SNX17 | 200991_s_at | 0.043114 |

| | | | | | | | | |
|---|---|---|---|---|---|---|---|---|
| SPIN2A /// SPIN2B | 211704_s_at | 0.047537 | | | | TMX4 | 201580_s_at | 0.043114 |
| ZNF76 | 207494_s_at | 0.047583 | | | | RELA | 209878_s_at | 0.043114 |
| JUN | 201465_s_at | 0.047601 | | | | ANKRD36B | 220940_at | 0.043114 |
| IGL@ | 215214_at | 0.047648 | | | | IGHMBP2 | 31861_at | 0.043114 |
| TTC21B | 220064_at | 0.04766 | | | | SCARB2 | 201647_s_at | 0.0489 |
| KLHL7 | 220238_s_at | 0.048002 | | | | ZER1 | 202448_s_at | 0.0489 |
| DDB2 | 203409_at | 0.048038 | | | | DENND4B | 202860_at | 0.0489 |
| GOSR1 | 204630_s_at | 0.048454 | | | | PRKAR2A | 204842_x_at | 0.0489 |
| COX7A2 | 217249_x_at | 0.048499 | | | | POFUT1 | 212349_at | 0.0489 |
| NR4A1 | 202340_x_at | 0.048549 | | | | HEG1 | 212822_at | 0.0489 |
| CD38 | 205692_s_at | 0.048549 | | | | ZDHHC17 | 212982_at | 0.0489 |
| CXCR6 | 211469_s_at | 0.048612 | | | | TWF1 | 214007_s_at | 0.0489 |
| JUNB | 201473_at | 0.048731 | | | | TRAK1 | 214924_s_at | 0.0489 |
| MKNK2 | 218205_s_at | 0.048809 | | | | KIAA0754 | 215268_at | 0.0489 |
| AHDC1 | 205002_at | 0.049595 | | | | NOSIP | 217950_at | 0.0489 |
| LST1 | 214574_x_at | 0.049754 | | | | LUC7L | 220143_x_at | 0.0489 |
| DUSP10 | 215501_s_at | 0.049754 | | | | --- | 222334_at | 0.0489 |
| | | | | | | ZNF337 | 37860_at | 0.0489 |
| | | | | | | ACAA2 | 202003_s_at | 0.050016 |
| | | | | | | FANCA | 203805_s_at | 0.050016 |
| | | | | | | GRAP | 206620_at | 0.050016 |
| | | | | | | ZNF141 | 206931_at | 0.050016 |
| | | | | | | IL6ST | 211000_s_at | 0.050016 |
| | | | | | | GPR135 | 211659_at | 0.050016 |
| | | | | | | AFF1 | 211826_s_at | 0.050016 |
| | | | | | | CKAP5 | 212832_s_at | 0.050016 |
| | | | | | | C14orf109 | 213246_at | 0.050016 |
| | | | | | | RABGAP1L | 215342_s_at | 0.050016 |
| | | | | | | --- | 215385_at | 0.050016 |
| | | | | | | LOC283079 | 215929_at | 0.050016 |
| | | | | | | --- | 216524_x_at | 0.050016 |
| | | | | | | DIAPH2 | 217246_s_at | 0.050016 |
