# Supplementary material for: Biomarker robustness reveals the PDGF network as driving disease outcome in ovarian cancer patients in multiple studies
Source: BMC Syst Biol. 2012 Jan 11;6:3. doi: 10.1186/1752-0509-6-3 (PMC3298526; doi:10.1186/1752-0509-6-3)
Supplement: Additional file 2 — Pathways Kaplan-Meier p-value. The table presents the pathways name and kaplan-meier log-rank p-value of all the significant pathways in the three datasets. [file 1752-0509-6-3-S2.PDF]

TCGA Dataset (511 patients)		Duke (119 patients)		Duke (42 patients)	
Pathway Name	Kaplan-Meier P value	Pathway Name	Kaplan-Meier P value	Pathway Name	Kaplan-Meier P value
PDGF signaling pathway(Biocarta)	0.007	PDGF signaling pathway(Biocarta)	0.001	PDGF signaling pathway(Biocarta)	0.01
glutathione metabolism(kegg)	0.000434	Ras signaling in the cd4+ TCR pathway(NCI/nature)	0.003	s1p1 pathway(NCI/nature)	0.012
glutamate metabolism(kegg)	0.000834	s1p1 pathway(NCI/nature)	0.005	Nongenotropic androgen signaling(NCI/nature)	0.021
ceramide signaling pathway(nci/nature)	0.001439	colorectal cancer(Kegg)	0.008	Ras signaling in the cd4+ TCR pathway(NCI/nature)	0.01
prostate cancer(kegg)	0.005402	retinol metabolism(Kegg)	0.009377	Aminosugars metabolism(Kegg)	0.024
ifn-gamma pathway(nci/nature)	0.008	starch and sucrose metabolism(Kegg)	0.012	Ceramide signaling pathway(Biocarta)	0.030681
valine, leucine and isoleucine degradation(kegg)	0.010	il-2 receptor beta chain in t cell activation(Biocarta)	0.033	colorectal cancer(Kegg)	0.043
ctcf: first multivalent nuclear factor(biocarta)	0.010582	Granzyme a mediated apoptosis pathway(Biocarta)	0.034	overview of telomerase rna component gene hterc transcriptional regulation(Biocarta)	0.05
granzyme a mediated apoptosis pathway(biocarta)	0.010825	Melanocyte development and pigmentation pathway(Biocarta)	0.034	erk1/erk2 mapk signaling pathway(Biocarta)	0.05
downregulated of mta-3 in er-negative breast tumors(biocarta)	0.010938	Nongenotropic androgen signaling(NCI/nature)	0.034		
selenoamino acid metabolism(kegg)	0.01297	role of egf receptor transactivation by gpcrs in cardiac hypertrophy(Biocarta)	0.034		
epithelial cell signaling in helicobacter pylori infection(kegg)	0.01607	vegfr3 signaling in lymphatic endothelium(NCI/nature)	0.037		
fructose and mannose metabolism(kegg)	0.01655	endothelins(NCI/nature)	0.04		
il 4 signaling pathway(biocarta)	0.01754	erk1/erk2 mapk signaling pathway(Biocarta)	0.043		
thyroid cancer(kegg)	0.017595	aminosugars metabolism(Kegg)	0.045		
valine, leucine and isoleucine biosynthesis(kegg)	0.018289	ccr3 signaling in eosinophils(Biocarta)	0.05		
igf-1 signaling pathway(biocarta)	0.018502				
chronic myeloid leukemia(kegg)	0.018651				
benzoate degradation via coa ligation(kegg)	0.019295				
pdgfr-alpha signaling pathway(nci/nature)	0.019372				
caspase cascade in apoptosis(biocarta)	0.0194				
fgf signaling pathway(nci/nature)	0.021867				
riboflavin metabolism(kegg)	0.028103				
inhibition of cellular proliferation by gleevec(biocarta)	0.028551				
cyanoamino acid metabolism(kegg)	0.034063				
alpha-synuclein and parkin-mediated proteolysis in parkinson`s disease(biocarta)	0.037912				
overview of telomerase protein component gene htert transcriptional regulation(biocarta)	0.039599				
ppar signaling pathway(kegg)	0.041409				
bone remodeling(biocarta)	0.042795				
limonene and pinene degradation(kegg)	0.044216				
