# Supplementary material for: Biomarker robustness reveals the PDGF network as driving disease outcome in ovarian cancer patients in multiple studies
Source: BMC Syst Biol. 2012 Jan 11;6:3. doi: 10.1186/1752-0509-6-3 (PMC3298526; doi:10.1186/1752-0509-6-3)
Supplement: Additional file 3 — FOS-JUN Correlation. The table presents the correlations between FOS and JUN, which are eventually the pathways output, in the two survival group. Group1, which correlates with higher survival rates, shows stronger correlation between the genes. [file 1752-0509-6-3-S3.PDF]

	TCGA (511 Patients)		Duke (119 patients)		Duke (42 Patients)	
	Rho	P value	Rho	P value	Rho	P value
Group1	0.57	5.297×10^{-6}	0.67	0.000001	0.62	0.001
Group2	0.4	6.26E-05	0.41	0.0004	0.42	0.024
