# Supplementary material for: Biomarker robustness reveals the PDGF network as driving disease outcome in ovarian cancer patients in multiple studies
Source: BMC Syst Biol. 2012 Jan 11;6:3. doi: 10.1186/1752-0509-6-3 (PMC3298526; doi:10.1186/1752-0509-6-3)
Supplement: Additional file 4 — Pathway calculation algorithm pipeline. The figure describes the calculation steps performed by the PathOlogist algorithm, starting with the RMA gene-expression levels. [file 1752-0509-6-3-S4.PDF]

RMA Expression levels

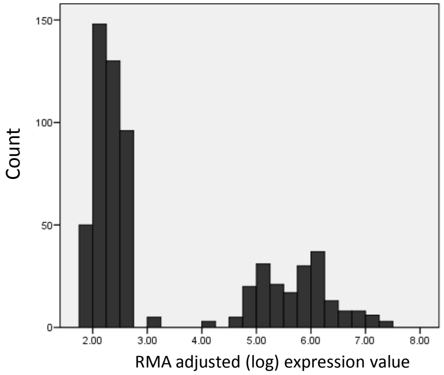

Up **D**own **P**robabilities are calculated from the RMA levels

$$p(x,Up) = p(Up) \times f(x \mid a_U, b_U) = \frac{N_U}{N} \times \frac{1}{b_U \Gamma(a_U)} X^{a_U - 1} e^{-\frac{x}{b_U}}$$

$$p(x,Down) = p(Down) \times f(x \mid a_D, b_D) = \frac{N_D}{N} \times \frac{1}{b_D \Gamma(a_D)} X^{a_D - 1} e^{-\frac{x}{b_D}}$$

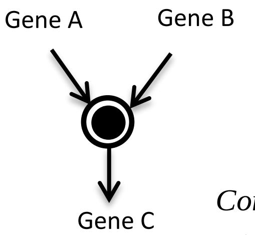

Activity

$$Interaction\_Activity = P(GeneA) \times P(GeneB)$$

Consistency

$$Consistency = P(active\_Interaction) \times P("Up"\_output\_gene) + P(inactive\_Interaction) \times P("Down"\_output\_gene)$$

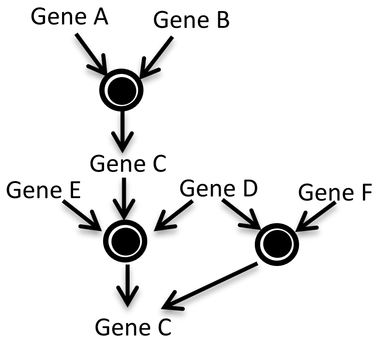

$$Pathway\_Activity = Average(InteractionA + \ldots + InteractionN)$$

$$Pathway\_Consistency = Average(InteractionA + \ldots + InteractionN)$$
